# Revisiting the direct and indirect impacts of enteric methane using a One Health perspective

**DOI:** 10.1093/af/vfaf010

**Published:** 2025-10-14

**Authors:** Eleanor May Pressman, Ermias Kebreab

**Affiliations:** Department of Animal Science, University of California, Davis, Davis, CA 95616; Department of Animal Science, University of California, Davis, Davis, CA 95616

**Keywords:** agriculture, climate change, enteric methane, feed additive, greenhouse gas, tropospheric ozone

**Implications**

We review not only the direct impacts of enteric methane on the environment but also the indirect impacts of enteric methane on human and animal health.
Enteric methane is a potent greenhouse gas, and climate change driven in part by methane emissions is expected to profoundly affect environmental, human, and animal health.
Methane is a precursor of tropospheric (surface-level) ozone, which is an air pollutant that damages crops and poses risks to human health.
Major advances have been made in developing tools to reduce enteric methane, but some strategies may have indirect effects that offset their net emissions reductions.
Future work should focus on quantifying the contribution of livestock agriculture to tropospheric ozone and assessing tradeoffs under methane-inhibition strategies to evaluate the impacts of methane on all One Health domains.

## Introduction

**Figure 1.**
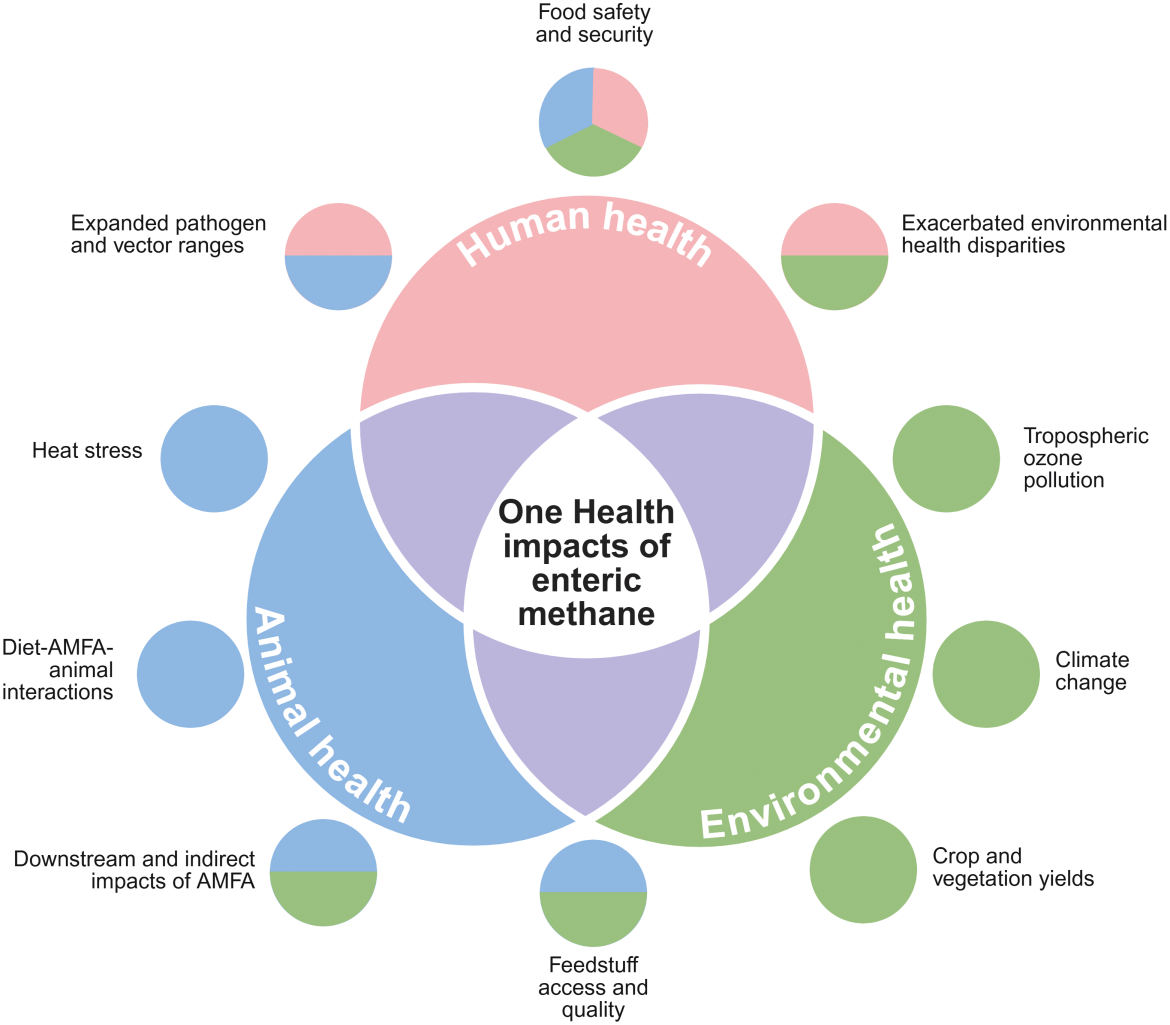
Illustration of the impacts of enteric methane on the One Health triad (animal, environmental, and human health domains). Most impacts noted are indirect impacts of methane via its direct impact on climate change or ozone, as discussed herein. Circles outside of the Venn diagram provide examples of impacts within each domain or spanning several domains; the colors of these circles correspond to the color of the domain(s) of impact in the Venn diagram. AMFA: antimethanogenic feed additive. Created in BioRender. Pressman, E. (2025) https://BioRender.com/u02e666.

Animal scientists face the challenge of balancing food security for a growing global population, addressing food safety concerns such as antimicrobial resistance and zoonotic disease outbreaks, and mitigating climatic change driven by greenhouse gas (GHG) emissions and land use change. The One Health framework emphasizes the interconnectedness of human, animal, and environmental health, advocating for multidisciplinary approaches to address pressing scientific problems. While animal agriculture is increasingly examined through a One Health lens (Nguyen et al., 2023; Verkuijl et al., 2024), enteric methane (CH_4_) —a key example of animal–environment interaction—is often narrowly viewed as an environmental issue related to climate change, with less focus on its indirect implications for human and animal health. This review explores the direct impacts of enteric CH_4_ on the environment, its indirect impacts on animal and human health via climate change and ozone pollution, and environmental tradeoffs associated with reducing enteric CH_4_ emissions from ruminants, highlighting the interconnectedness of research advances and challenges across these domains (Figure 1).

## Impacts of Enteric CH_4_ on the Environment

The direct environmental impacts of enteric CH_4_ are well-documented. Livestock agriculture contributes to GHG emissions through enteric fermentation, manure management (producing nitrous oxide (N_2_O) and CH_4_), and land use change. Ruminant production accounts for about a third of global annual CH_4_ emissions (Saunois et al., 2016). CH_4_ is the second most impactful agriculturally relevant anthropogenic GHG after CO_2_ and total global CH_4_ is responsible for about 0.5 °C of the 1.1 °C of human-induced global warming and about 20% of the net radiative forcing increase since the preindustrial era (Myhre et al., 2013; IPCC, 2021). These increases in radiative forcing threaten the stability and resilience of the Earth’s climate system (Richardson et al., 2023).

Methane is also a major precursor of tropospheric (surface-level) ozone (O_3_), a potent GHG and air pollutant that damages crops and reduces vegetation yields. Ozone’s negative impacts on crop productivity may exacerbate threats to global food and feed production caused by climate change under some scenarios (Tai et al., 2014) while also impeding CO_2_ uptake by plants, further intensifying climate change (Sitch et al., 2007). Methane accounts for 35% of tropospheric O_3_ formation (Butler et al., 2020), though the specific contribution of enteric CH_4_ to global surface-level O_3_ remains unclear.

## Indirect Impacts of Enteric CH_4_ on Animal Health

Enteric CH_4_ indirectly impacts animal health by contributing to climate change. Climate change poses direct and indirect threats to livestock health and welfare, including heat stress, reduced feed availability, and expanded pathogen and vector ranges (Lacetera, 2019). Enteric CH_4_ mitigation strategies can also focus directly on animal health and welfare, such as improving nutrition to meet genetic potentials for milk or beef production, improving herd reproductive management, and mitigating disease burdens (Hristov et al., 2013). These strategies aim to enhance production efficiency, thereby reducing CH_4_ emissions per unit of animal product (emissions intensity).

Recent advancements in antimethanogenic feed additives (AMFA) offer tools to reduce enteric CH_4_ emissions by modifying the rumen environment or by directly inhibiting methanogenesis. However, a One Health framework underscores the need to evaluate the downstream and indirect impacts of AMFA use. For example, the extensively studied AMFA 3-nitrooxypropanol (3NOP) decreases enteric CH_4_ emissions by up to 40% in dairy cattle, yet achieving carbon neutrality in the dairy industry requires reductions exceeding 50% (Beauchemin et al., 2024). While higher grain diets may enhance 3NOP’s efficacy (Kebreab et al., 2023), grain production, deforestation for cropland expansion, and 3NOP manufacturing could contribute to indirect production of GHG emissions (Feng and Kebreab, 2020).

Methane-inhibition often increases hydrogen (H_2_) emissions, which can offset net emissions reductions due to H_2_’s indirect radiative forcing effects, although this impact may be minor (Hristov and Solomon, 2025). Additionally, AMFA supplementation may alter emissions of N_2_O, a potent GHG with a greater global warming potential than CH_4_. For example, manure from cows fed 3NOP has been shown to increase soil N_2_O emissions in certain soils (Weber et al., 2021), and nitrate supplementation may increase enteric N_2_O emissions (Petersen et al., 2015).

The cultivation of bromoform-containing red seaweed (*Asparagopsis*) or the production of synthetic bromoform-containing AMFA raises concerns about stratospheric ozone depletion if not managed properly. While current evidence suggests minimal ozone-depleting effects from *Asparagopsis* aquaculture (Jia et al., 2022), the impacts of synthetic bromoform production remain unexplored. A comprehensive evaluation of AMFA within a One Health framework is essential to ensure net benefits for environmental and animal health.

## Indirect Impacts of Enteric CH_4_ on Human Health

Given its considerable contribution to climate change, enteric CH_4_ from ruminant livestock indirectly impacts human health, as climate change poses a profound threat to public health. On the other hand, animal agriculture plays a critical role in human health by providing nutrient-dense foods that combat malnutrition and nutrient deficiencies, especially in the developing world (Randolph et al., 2007). Global demand for animal-source foods is expected to rise by between 61% and 144% to feed the predicted human population of nearly 10 billion by 2050 (Valin et al., 2014), with a concomitant rise in the global ruminant herd size (Thornton, 2010). Mitigation of the CH_4_ emissions of this growing ruminant population is essential to balance global food security and climate impact.

Enteric CH_4_ also indirectly impacts human health as a precursor of tropospheric O_3_, which is associated with premature mortality. Because CH_4_ is a well-mixed, short-lived GHG, reducing CH_4_ emissions is an effective strategy to simultaneously curb global temperature rise in the short term and reduce O_3_-related health impacts. West et al. (2006) estimated that a 20% reduction in global anthropogenic CH_4_ emissions in 2010 could prevent 370,000 premature O_3_-associated deaths globally from 2010 to 2030 while also reducing both CH_4_- and O_3_-induced radiative forcing. Importantly, because CH_4_ is longer-lived in the atmosphere than other volatile O_3_ precursors, local CH_4_ emissions affect global surface-level O_3_ concentrations, meaning that curbing local CH_4_ emissions would reduce O_3_ concentrations by about the same amount in rural and urban areas (West et al., 2006). However, the health impacts of O_3_ pollution are not evenly distributed. Rural populations are exposed to higher O_3_ levels than urban residents (Sun et al., 2024), and the effects of transboundary O_3_ transport vary by country income-levels (Chen et al., 2022). Addressing these environmental health inequities is critical to understanding the broader impacts of enteric CH_4_ within the One Health framework.

## Conclusions

Considerable progress has been made over the past decade in developing nutritional, genetic, and management interventions to reduce enteric CH_4_ emissions from ruminant livestock, demonstrating the success of collaborative research at the animal–environment nexus. Future work should further explore biosphere–atmosphere interactions through interdisciplinary research (e.g., the Carbon-I Phase A concept study, https://carbon-i.github.io/), to better attribute tropospheric O_3_ pollution to livestock CH_4_ emissions. Continued evaluation of the net environmental impacts of AMFA is also key as adoption of these tools becomes more widespread. This review highlights the impacts of enteric CH_4_ on animal, human, and environmental health within a One Health framework, emphasizing its central role in addressing these interconnected challenges.

## About the Authors

**Eleanor Pressman** is a dual DVM-PhD candidate in the Veterinary Scientist Training Program at the University of California, Davis. She is currently conducting her graduate research, which focuses on evaluating antimethanogenic feed additives and predicting their efficacy using mechanistic modeling, in the Department of Animal Science under the mentorship of Dr Ermias Kebreab. **Corresponding author:**  empressman@ucdavis.edu

**Ermias Kebreab** is a professor and an Associate Dean for Global Engagement at the University of California, Davis, where he holds the Sesnon Endowed Chair position. He has worked around the world on improving livestock systems to be more sustainable. Dr Kebreab holds BSc degrees in biology with minor in chemistry, and a PhD in integrated biology. His research focuses on reducing the environmental impact of livestock—especially greenhouse gas emissions such as methane—through better nutrition and innovative solutions including feed additive and genetic engineering. He uses computer models and real-world experiments to find solutions that work for farmers and the planet. His work has been influential in shaping international climate and livestock policy. He is widely recognized for his leadership in the field of sustainable animal agriculture.
